# Automated assessment of foot elevation in adults with hereditary spastic paraplegia using inertial measurements and machine learning

**DOI:** 10.1186/s13023-023-02854-8

**Published:** 2023-08-29

**Authors:** Malte Ollenschläger, Patrick Höfner, Martin Ullrich, Felix Kluge, Teresa Greinwalder, Evelyn Loris, Martin Regensburger, Bjoern M. Eskofier, Jürgen Winkler, Heiko Gaßner

**Affiliations:** 1 Department of Molecular Neurology, University Hospital Erlangen, Friedrich-Alexander-Universität Erlangen-Nürnberg, Schwabachanlage 6, Erlangen, 91054 Germany; 2 Machine Learning and Data Analytics Lab, Department of Artificial Intelligence in Biomedical Engineering (AIBE), Friedrich-Alexander-Universität Erlangen-Nürnberg, Erlangen, Germany; 3 Center for Rare Diseases Erlangen (ZSEER), Universitätsklinikum Erlangen, Erlangen, Germany; 4 Fraunhofer IIS, Fraunhofer Institute for Integrated Circuits IIS, Erlangen, Germany

**Keywords:** Gait analysis, Wearable sensors, Classification, Range of motion, Motion capture, Muscle spasticity

## Abstract

**Background:**

Hereditary spastic paraplegias (HSPs) cause characteristic gait impairment leading to an increased risk of stumbling or even falling. Biomechanically, gait deficits are characterized by reduced ranges of motion in lower body joints, limiting foot clearance and ankle range of motion. To date, there is no standardized approach to continuously and objectively track the degree of dysfunction in foot elevation since established clinical rating scales require an experienced investigator and are considered to be rather subjective. Therefore, digital disease-specific biomarkers for foot elevation are needed.

**Methods:**

This study investigated the performance of machine learning classifiers for the automated detection and classification of reduced foot dorsiflexion and clearance using wearable sensors. Wearable inertial sensors were used to record gait patterns of 50 patients during standardized 4 $\times$ 10 m walking tests at the hospital. Three movement disorder specialists independently annotated symptom severity. The majority vote of these annotations and the wearable sensor data were used to train and evaluate machine learning classifiers in a nested cross-validation scheme.

**Results:**

The results showed that automated detection of reduced range of motion and foot clearance was possible with an accuracy of 87%. This accuracy is in the range of individual annotators, reaching an average accuracy of 88% compared to the ground truth majority vote. For classifying symptom severity, the algorithm reached an accuracy of 74%.

**Conclusion:**

Here, we show that the present wearable gait analysis system is able to objectively assess foot elevation patterns in HSP. Future studies will aim to improve the granularity for continuous tracking of disease severity and monitoring therapy response of HSP patients in a real-world environment.

## Background

Hereditary spastic paraplegias (HSPs) are a group of rare inherited diseases with a prevalence of less than 10/100,000 [1]. Common for all subtypes of HSP, a slowly progressing paresis and spasticity of the legs leads to a characteristic gait pattern. In the lower leg, increased muscle tone of plantar flexors and paresis of dorsal extensors lead to an increased plantar flexion [2]. This results in a reduction of range of motion (RoM) and foot clearance. Consequently, patients experience an increased risk of stumbling or even falling [3]. Antispastic therapy includes both strengthening and stretching of muscles, as well as medication to reduce muscle tone, e.g. botulinum toxin [4, 5]. Disease severity and progression, including leg spasticity, are commonly assessed using clinical rating scales. However, the Modified Ashworth scale (MAS), Modified Tardieu scale (MTS) and the most commonly applied Spastic Paraplegia Rating Scale (SPRS) do not quantify spasticity while the patient is walking. [6–8]. Consequently, information about the effect of leg spasticity and paresis on RoM and foot clearance during walking is not assessed and crucial information in regard to the risk of stumbling and falling is missing. Active motion in terms of gait is only part of the SPRS with the items ’walking distance without pause’, ’maximum gait speed’, and ’gait quality’. However, these parameters do not directly assess RoM and foot clearance while walking. Additionally, the widely used clinical rating scales may be susceptible to patient- and clinician-dependent variability [9–11]. For the SPRS, it was observed that it does not capture small changes in disease severity. Therefore, disease-specific digital biomarkers are required in order to quantify changes with a finer granularity [12]. Furthermore, the necessity of a trained specialist for performing clinical rating scales limits their applicability, such that symptom fluctuation or disease progression is difficult to longitudinally capture in patients’ everyday life.

Several studies have shown that gait parameters obtained from optical motion capture systems are relevant in HSP and thus may be important as disease-specific digital biomarkers [12–17]. The most often reported relevant parameters are RoM of ankle, knee, and hip joints [13–15] and foot clearance [14, 15]. Serrao et al. [13] analyzed the gait of 50 HSP patients. They reported statistically significant differences regarding ankle RoM, and thus reduced foot dorsiflexion, between subgroups of patients with different disease severities and controls. These results were recently confirmed in a study by Laßmann et al. [14], who analyzed the gait of 47 HSP patients and 23 controls. Besides confirming differences in ankle RoM between groups of HSP patients and healthy controls, they detected significantly lower foot clearance in HSP patients. Based on this study, the reduction of foot elevation, i.e. ankle RoM and foot clearance, is even apparent at early disease stages when a clinical rating scale did not detect changes. Therefore, this suggests a higher sensitivity of instrumented gait analysis than state-of-the-art clinical rating scales. Additionally, the granularity is higher, since optical motion captures systems offer metrics with sub-millimeter accuracy. The higher sensitivity and finer granularity show the relevance of instrumentally assessed gait parameters as digital biomarkers for HSP. Although studies with optical motion capture systems provide important insights into the gait parameters of HSP patients, the major shortcoming of these systems is that they cannot be used to assess patients’ gait continuously in their everyday life.

In contrast to optical motion capture systems, wearable sensors enable gait analysis for HSP patients in unconstrained environments [16–19]. Studies with different wearable inertial measurement units (IMUs) showed technical validity and repeatability of recorded gait parameters [18, 19]. However, according to Coccia et al. [19], knee RoM measured with wearable sensors is not robust enough to distinguish HSP patients from healthy controls. Next to these technical studies, the clinical relevance of gait parameters has also been demonstrated. A study including 112 HSP patients reported significant associations of gait parameters with different items of the SPRS, such as impaired mobility and disease duration [17]. Furthermore, the gait parameters correlate to fear of falling and the self-perceived quality of life of HSP patients [16].

Previous work showed that gait parameters reflect clinically relevant information, such as the SPRS or quality of life, in HSP [12–17]. However, there is an urgent need to capture HSP gait cycles using an automated and continuous assessment of phenotypical disease characteristics. Therefore, this study aimed to investigate the automated assessment of phenotypical disease characteristics in terms of reduced foot elevation from wearable sensor data. More precisely, we aimed to develop an algorithm to (1) detect reduced RoM and foot clearance and (2) classify the severity of this reduction in HSP patients. This type of algorithm is able to tremendously increase the clinical utility of instrumented gait analysis and is the first step toward continuous instrumented tracking of disease-specific gait patterns in HSP patients.

## Methods

### Patients and assessments

Fifty HSP patients were enrolled in the study during their outpatient visits in the Movement Disorders Outpatient Unit at the University Hospital Erlangen, Germany. Eligible patients had a genetic or clinical diagnosis of spastic paraplegia and were able to walk at least 10 ms. The study was approved by the local ethics committee Nr. 4208 (21.4.2010) / Nr. 166_18 B (25.05.2018), and written informed consent was obtained from all patients according to the Declaration of Helsinki.

The deficits of gait parameters were heterogeneously distributed among the patients enrolled. While most patients (N = 29) were able to walk independently, eleven patients used walking sticks or crutches, and ten patients used support from another person or a wheeled walker. The heterogeneity of the patient cohort is further reflected by a wide range of disease duration (< 1 to 41 years) and disease-specific symptoms based on the SPRS (Table 1). Most importantly in terms of this study, the patients presented diverse severities of reduced ankle RoM and foot clearance.

**Table 1:** Patient characteristics. SPRS: Spastic Paraplegia Rating Scale

Demographic / clinical feature	N = 50
Sex (n female/ n male)	26 / 24
Age (years)	48 ± 15 (19 to 71)
Genotype (n)	
SPG4	18
unknown	11
SPG11	8
SPG7	6
SPG3/10/15/39 or GDAP1	5
SPG5	2
Presentation (n pure / n complex)	28 / 22
Disease duration (years)	14 ± 10 (<1 to 41)
SPRS	19 ± 8 (4 to 35)
Walking aids	
None	29
Walking sticks / crutches	11
Arm of relative / supervisor	5
Wheeled walker	5

Out of the five patients using a wheeled walker, two used a wheelchair as a wheeled walker. Age, disease duration, and SPRS are reported as mean ± standard deviation (minimum to maximum)

### Data acquisition and annotation

Patients performed a standardized 4x10m gait test at a self-chosen speed to assess the reduction of foot elevation while walking. The gait tests were conducted in a laboratory environment under the supervision of a movement disorder specialist. For the assessment, shoes were equipped with two Shimmer 2R/3 IMUs attached laterally to each shoe [18]. These devices recorded three-dimensional acceleration ($m/s^2$) and gyroscopic rate ($^\circ /s$) signals at 102.4 Hz. Additionally, the gait tests were recorded on videos for later clinical annotation.

**Fig. 1:**
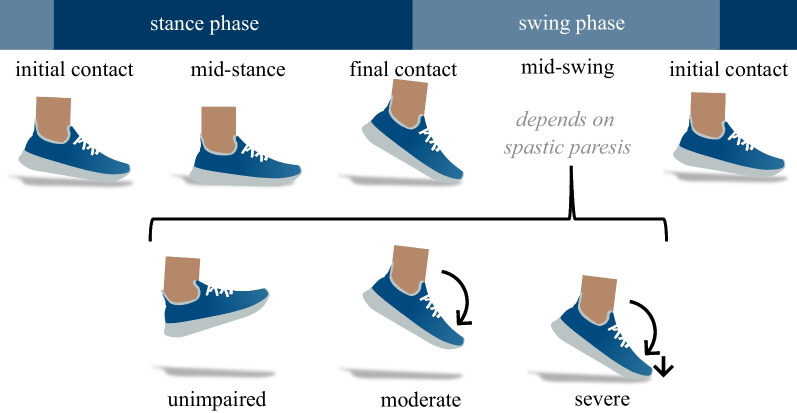
Schematic representation of a stride. If the angle between shin and foot was increased during mid-swing, this was defined as a *moderate* impairment. If additionally the foot touched the ground during the swing phase, this was annotated as *severe* impairment

Three movement disorder specialists diagnosed qualitative disease-specific clinical annotations regarding the reduction of foot elevation. Each annotator rated each of the patients’ feet separately since severity was not always symmetrical. They assigned one of three classes, which were specifically designed for this study based on two clinically relevant observations during the swing phase (Fig. 1). When foot dorsiflexion was intact, annotators assigned *unimpaired* impairment. Increased plantarflexion / reduced ankle RoM with a potential decrease in foot clearance but the foot not touching the ground during the swing phase was annotated as *moderate* impairment. *Severe* impairment was defined as a combination of increased plantarflexion and the foot touching the ground one or several times during the swing phase. These classes were defined based on previous studies [13–15]. After three movement disorder specialists had separately annotated the dataset, the majority vote was used to generate ground truth labels for each foot. Thus, for each of the 50 patients, one annotation per foot was obtained, resulting in 100 samples. To address the detection of *reduced RoM and foot clearance* in a binary classification, the classes *moderate* and *severe* were fused to the class *impaired*. For the classification of severity in a multiclass classification, the three class labels *unimpaired*, *moderate*, and *severe* were used.

### Algorithm

**Fig. 2:**
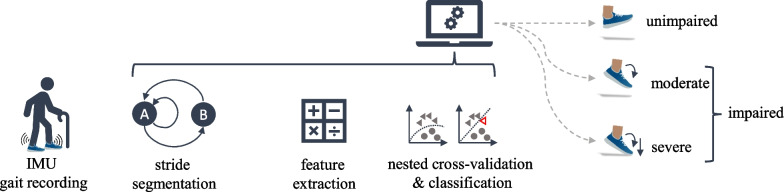
Schematic representation of the evaluation pipeline. IMU: Inertial measurement unit

The algorithm pipeline used in this study consists of three parts (Fig. 2). We first segmented the gait signal into single strides. For each stride, we calculated features from several domains. Each feature describes a characteristic part of the stride, for example stride duration or number of peaks in the signal. These features along with the previously generated annotations were used to analyze the performance of four different classifiers in a leave-one-participant-out (LOPO) cross-validation (CV). For this purpose, a participant was excluded from the dataset and the classifier was trained on the remaining 49 participants. Then, the classifier was used to predict the severity for the left-out participant. This was repeated for each participant, such that a prediction for all participants was obtained and the classifier’s performance could be assessed.

For classifiers necessitating a fitting based on hyperparameters, we used a nested cross-validation [20]. All classifiers were trained and analyzed separately for a two-class (binary) and a three-class (multiclass) classification.

#### Stride segmentation

The IMU data was segmented into single strides to prepare the feature extraction on a stride level. Stride segmentation was performed according to a previously developed algorithm for gait analysis in HSP [18]. It uses a Hidden Markov Model (HMM) to predict swing and stance phases from the IMU data. For this study, consecutively predicted stance and swing phases were fused to a stride. Labels for stance not having an immediately following swing label were discarded, and vice versa for swing labels. Turning strides were manually excluded from the analysis in this study using a graphical user interface [21] with a custom plugin, as the HMM was developed and validated exclusively for straight strides.

#### Feature extraction

After segmenting data into single strides, a total of 21 features based on literature, developed by movement disorder specialists in this study, and generated from the HMM were extracted. To model the ankle RoM, we calculated the trapezoidal integral of the gyroscope data around the mediolateral axis, which mirrors plantar flexion and dorsal extension. By integrating the gyroscopic rate ($^\circ /s$) with respect to time, we obtained the angle between IMU and floor at any given time during the gait cycle. Then, RoM was assessed as the difference between the maximum and minimum of this integral. In a cohort of patients with L5-radiculopathy, Bidabadi et al. [22] suggested assessing the number of negative peaks in the gyroscope signal around the mediolateral axis. According to their analysis, a negative peak before the swing phase is only present if patients have intact dorsiflexion. Furthermore, we calculated the integral and difference between the maximum and minimum for the L2-norm ($\sqrt{x^2 + y^2 + z^2}$) of the 3D gyroscope and acceleration data. We derived further signal processing features from the autocorrelation of the normed acceleration and gyroscopic rate: the difference between maximum and minimum, number/width of peaks, and width of the highest peak in the signal. As temporal features, swing and stance times were calculated from the labels generated by the HMM. Furthermore, we extracted maximum swing velocity, as well as minimal and maximal gyroscopic rate as features after discussing the gait characteristics of impaired patients within an interdisciplinary team of medical experts, a sport scientist, and engineers.

#### Classification

We conducted several classification experiments using the features calculated for each of the dataset’s 3855 strides as input vectors and the annotations as ground truth labels for *unimpaired*, *moderate*, or *severe* reduction of foot elevation. When using all three classes as given by the majority vote of annotations, this is referred to as multiclass classification in this study. For binary classification, the labels *moderate* and *severe* were joined to a label *impaired*, as mentioned above.

The most straightforward approach, classifying RoM signals based on thresholds, was based on literature suggesting that RoM of the ankle may be a suitable parameter to classify different groups of HSP patients [13] and foot drop patients [22]. For this approach, RoM was calculated as described in the section *Feature extraction*. A threshold classifier was set up with one and two thresholds for the binary and multiclass classification, respectively. The thresholds were optimized in the LOPO in terms of the balanced accuracy as implemented in scikit-learn [20]. Furthermore, we evaluated a Gaussian Naive Bayes classifier in a LOPO.

For two additional classifiers, Support Vector Machine (SVM) and Random Forest (RF), optimization of hyperparameters was necessary. These parameters define various aspects of the classifier, including the complexity of the decision boundary or regularization strength. Selecting hyperparameters is crucial as it impacts the classifier’s ability to capture important aspects of the training data while avoiding overfitting. The hyperparameter spaces, which specify the ranges explored during optimization, are listed in Table 2. To determine the optimal parameters, a five-fold cross-validation for ten randomly selected parameters was performed on each training set.

Additionally, we performed a feature selection in the inner CV using recursive feature elimination with a decision tree with balanced class weights as the estimator. The number of features was set to the square root of the number of patients in the LOPO ($\sqrt{50-1}=7$) following the scikit-learn default value when fitting random forests. For the SVM classifier, features were normalized in the inner CV to zero-mean and unit variance. After the inner CV, the classifier was refitted using the optimal hyperparameters on 49 participants. The predictions on the outer left-out participant’s strides were aggregated to a single prediction per foot by a majority vote. These majority votes were compared with the majority vote of all annotators using the balanced accuracy score.

**Table 2:** Search spaces for (hyper-)parameters of classifiers

Classifier	Parameter	Values
Threshold range of motion	threshold in degrees	{0, 1, 2, ..., 120}
Support vector machine	C	{0.1, 1, 10, 100, 1000, 10000}
Random forest	Number of trees	{100, 500, 1000}
	Minimum number of samples per leaf	{20, 40, 60, 80, 100, 120, 140}

## Results

For the recorded dataset, including 100 samples (feet), all three movement disorder specialists assigned the same class in 47% (total agreement). Out of the remaining 53%, only for one foot, all specialists disagreed and suggested a different severity. They reassessed this sample together and jointly assigned it to the class *moderate*. Overall, according to the majority vote, the classes *unimpaired* and *moderate* reduction of foot elevation had an equal size (Table 3). Fewer feet samples were assigned to the class *severe* reduction of foot elevation impairment.

Concerning the majority vote, individual specialists reached an agreement of 77%, 83%, and 86% (average 82%). For the binary classification, the average agreement increased to 88%. Consequently, we aimed at an accuracy of the developed algorithms of 82% for the multiclass and 88% for the binary classification task. Reaching these values would indicate an equal performance of the algorithm with a movement disorder specialist.

**Table 3:** Annotations were obtained using a majority vote of three movement disorder specialists

Severity	Labelled feet (left / right)
Unimpaired	37 (18 / 19)
Moderate	37 (20 / 17)
Severe	26 (12 / 14)

**Fig. 3:**
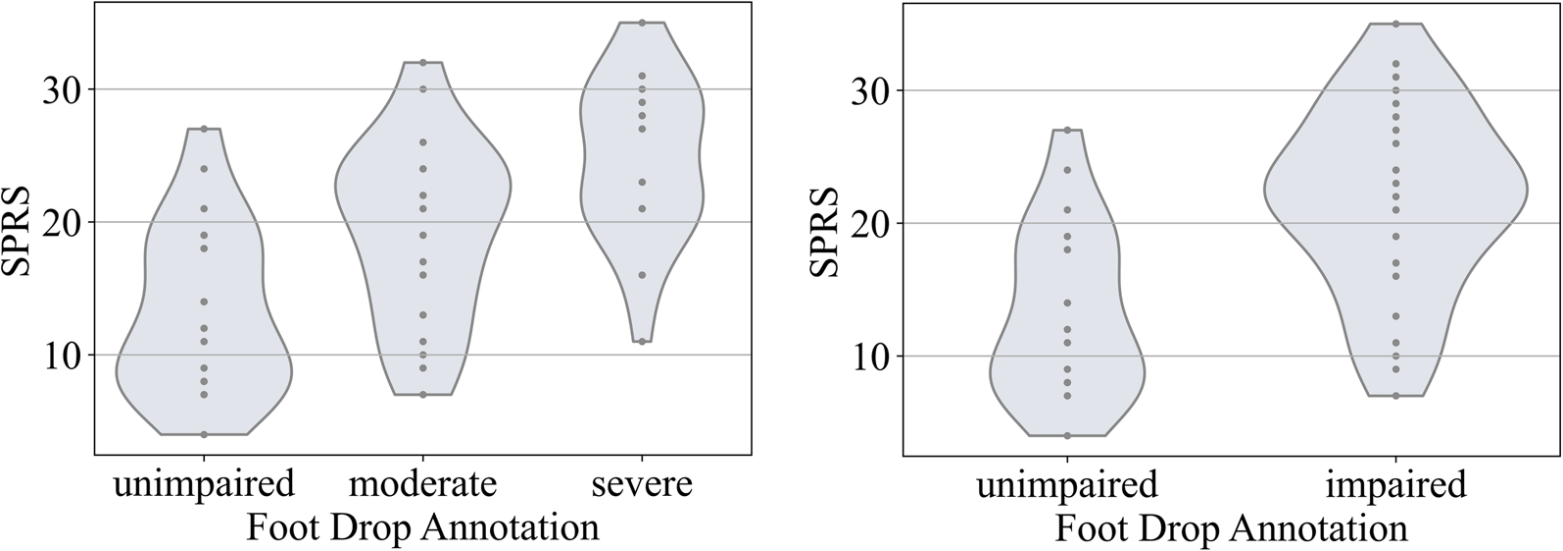
Distribution of Spastic Paraplegia Rating Scale (SPRS) values for different classes. Both images show the same dataset, in the right image the classes moderate and severe were fused to the class impaired

The relation between SPRS values and annotations is shown in Fig. 3. This shows an increase in SPRS with increased levels of reduction in foot elevation.

3855 strides were segmented algorithmically and used for data analysis. On average, 77.1 strides per patient were included, with a minimum of 46 and a maximum of 123 strides. For each classification task, binary and multiclass, we report two results (Table 4): While one result reflects the accuracy across all 100 samples, the other result focuses on the subset of samples with total agreement among all three annotators (N = 47). This results in four scenarios per classifier (binary/multiclass and all / total agreement samples only).

For both binary and multiclass classification, the threshold RoM classifier shows the lowest performance with an accuracy of 47–67% (Table 4). The Gaussian Naive Bayes has a higher accuracy of 62–75%. The best results were obtained using SVM and RF classifiers (71–87%). This corresponds to an improvement of more than 50% of the SVM and RF compared to the simple RoM threshold classifier for the multiclass classification. Both SVM and RF (86/87%) nearly reach the performance of a human annotator (88%) for binary classification. Exclusively analyzing samples with total agreement had a small effect on the binary classification using SVM and RF (89/87%.) For the multiclass scenario, SVM and RF (71/74%) do not reach the annotators’ accuracy (82%). However, the accuracy is remarkably higher when exclusively considering samples with total agreement among annotators. In this case, RF improved from 74 to 89% resulting in a similar performance as for the binary classification. The differences in accuracy between SVM and RF are negligible, except for the multiclass scenario exclusively analyzing samples with total agreement. In this scenario, the RF outperforms the SVM (89/78%).

**Fig. 4:**
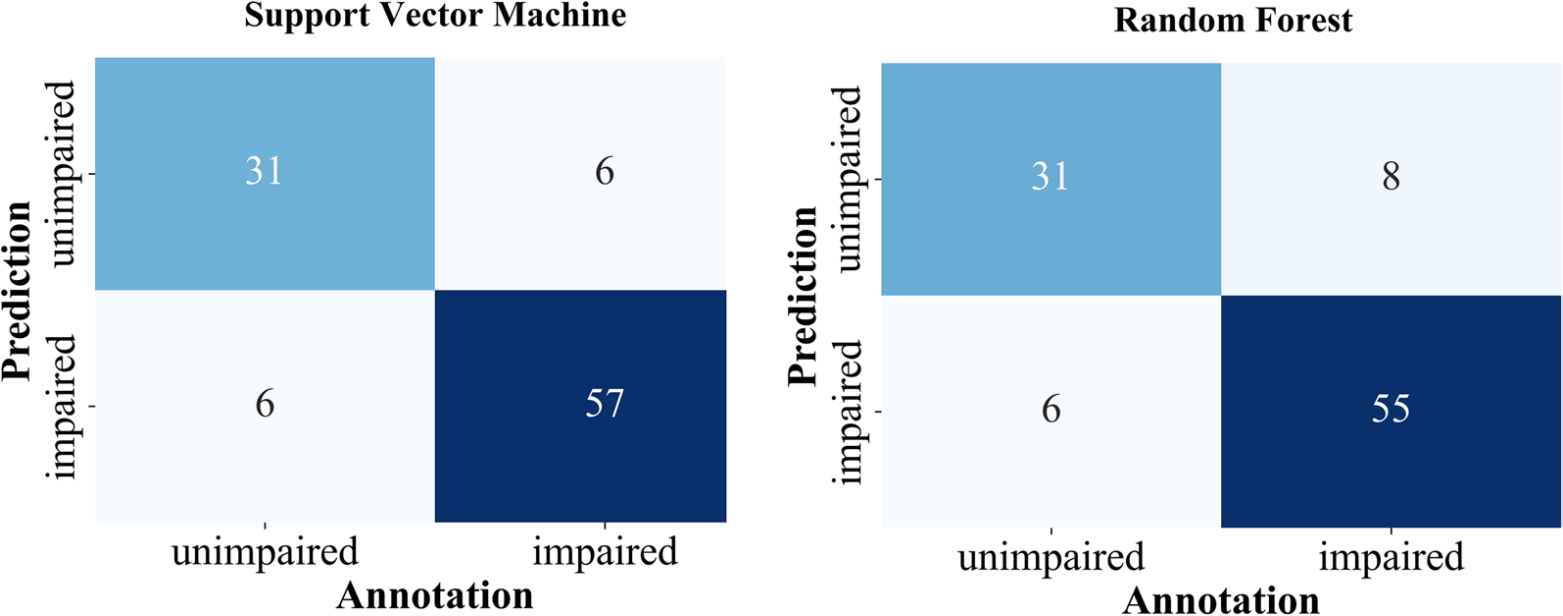
Confusion matrices for the binary classification. The class impaired contains all samples of the classes moderate and severe

**Fig. 5:**
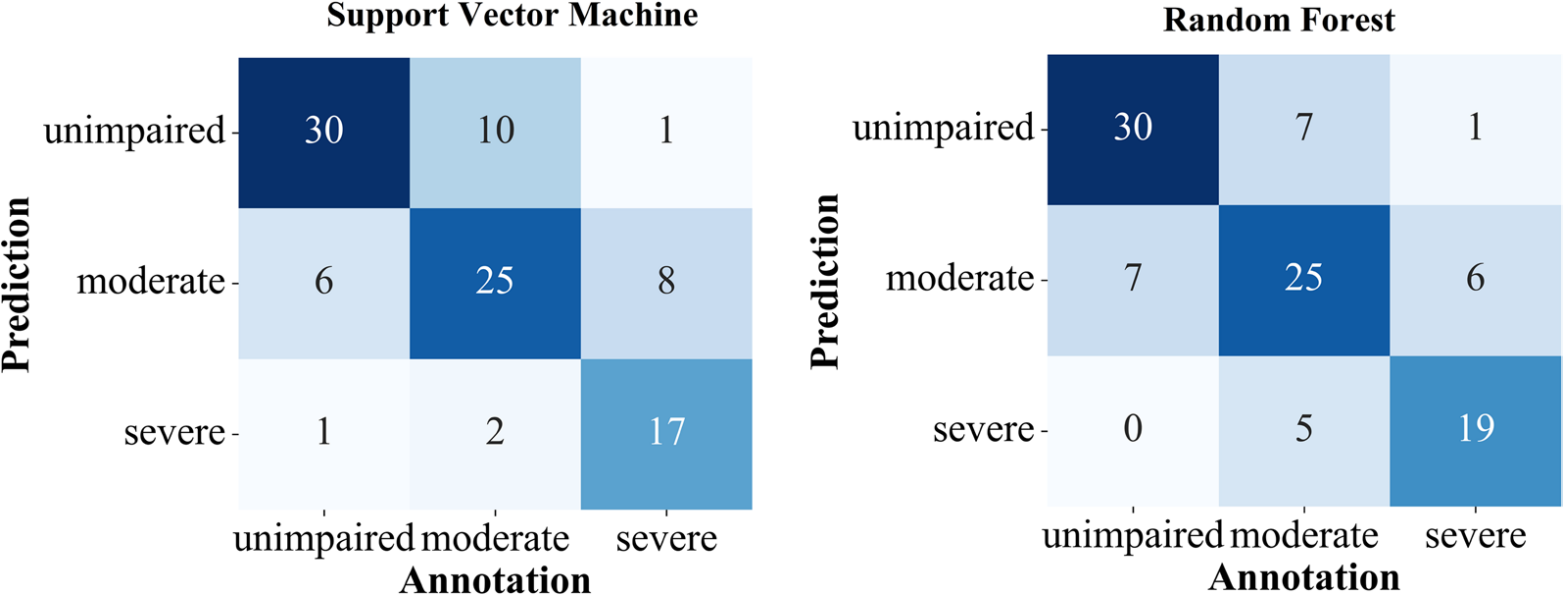
Confusion matrices for the multiclass classification

The error distribution was mostly balanced regarding over- and under-estimation of severity in reduced foot elevation (Figs. 4 and 5). However, the multiclass SVM approach tended to underestimate the severity (Fig. 5). In the case of binary classification, SVM and RF misclassified 12 and 15 samples, respectively. On average, 23% of these samples’ strides were correctly classified by SVM and RF. For multiclass classification, this value was slightly higher at 24% and 28% for RF and SVM, respectively.

**Table 4:** Balanced accuracy for trained classifiers (%)

Classifier	Binary	Multiclass
Threshold range of motion	67 (65)	47 (46)
Gaussian Naive Bayes	75 (71)	62 (67)
Support vector machine	87 (89)	71 (78)
Random forest	86 (87)	74 (89)
Annotators average	88 (100)	82 (100)

Values in brackets show the results for foot samples with total agreement among annotators (N = 47)

## Discussion

In this study, we analyzed whether reduced foot elevation in HSP patients is detected by machine learning algorithms using wearable sensor data recorded during supervised standardized gait tests. Additionally, we analyzed whether the severity of the impairment can be classified by machine learning algorithms. We compared classification accuracies for a thresholding algorithm, which was inspired by results from previous studies [13–15], with three machine learning algorithms. The comparison was based on data from 50 HSP patients annotated by three trained movement disorder specialists. Our results showed that SVM and RF are the most promising approaches for the detection and classification of reduced foot drop elevation in HSP patients during gait.

Our study extends the findings of previous studies. Serrao et al. detected significant differences in the range of motion of the ankle for patients with different disease severities. This is further supported by a study from Laßmann et al. additionally showing that foot clearance is a relevant gait parameter for HSP. Even though we used wearable sensors instead of an optical motion-capture system, our dataset reflects similar relations. The annotations show a positive relationship with the SPRS of patients, supporting the plausibility of our annotations (Fig. 3). Moreover, our study’s annotations indicate a wide range of phenotypical presentations among the patients in terms of reduced ankle RoM and foot clearance. This is evident from the fact that one annotator assigned a different severity level compared to the remaining two annotators in 53% of the cases. Regarding achieved accuracy, our results are in a similar range as reported in the domain of foot drop caused by L5-radiculopathy. While Bidabadi et al. [23] reached a classification accuracy of 84%, we report an accuracy of up to 89% in a phenotypically very different and diverse cohort of HSP patients.

In contrast to previous literature, this study focussed on the algorithmic detection and classification of impaired foot elevation in HSP patients. Therefore, this approach is an important step for the development of qualitative, disease-specific gait parameters in HSP. However, the suggested approach currently has limited applicability and needs to be further developed before it can be applied in clinical practice. The annotators did not rely on a pre-existing clinical assessment but generated the annotations based on video recordings of walking patients. While relevant clinical assessments, such as SPRS or MAS, do assess calf spasticity or foot dorsiflexion, these are not performed while the patient is walking. Therefore, they do not necessarily reflect the level of impairment while walking. This is shown by the significant but weak to moderate correlation (r < 0.7) of gait parameters with the SPRS value [14, 17]. Therefore, we argue that our approach is suitable for this pilot study.

Notably, this pilot study with the three-class annotations does not allow tracking small changes in the severity of reduced foot elevation yet. In order to address this in a future study, we will apply an optical motion capture system for generating ground truth annotations. By this, the granularity of annotations will be increased. Additionally, using an optical motion capture system as ground truth will increase the objectivity of annotations. Since our results for the multiclass RF classification showed a large improvement when only using samples with a total agreement, we assume that increasing the objectivity in annotations will also improve the classification performance. Thus, using an optical motion capture system will improve granularity by training a regressor, and increase accuracy at the same time.

In the future, the algorithmic analysis of reduced foot elevation in HSP patients might be incorporated into the assessment of disease progression or evaluation of functional improvements induced by therapies such as botulinum toxin or physiotherapy. Furthermore, the algorithmic analysis may be able to provide the possibility for a closed-loop system, e.g. for functional electrical stimulation or focal muscle vibration, increasing foot clearance and therefore reducing the risk of stumbling or even falling [24, 25]. Consequently, patients’ walking deficits, which are a major burden [3], might be reduced. Another important research direction could be whether the classification is a suitable parameter to assess subjective gait impairments. Gait impairments in HSP vary between days resulting in a varying probability of stumbling and falling as known from a study by Kerstens et al. [3]. Therefore, a continuous measurement might reveal important results for the evaluation of functional capacity in patients’ everyday life.

## Conclusion

HSP-specific reduction in foot elevation may be detected automatically in standardized gait tests using wearable sensors and machine learning classifiers. The accuracy is close to those of clinical specialists for binary classification. For the classification of severity, accuracy is lower, however, might be improved in future studies. With the findings from this study, we reinforce the utility of instrumented gait analysis in HSP. We are convinced that this will ultimately help to develop continuous tracking of disease severity and therapy response of HSP patients in flexible environments including the real world.

## Data Availability

The dataset supporting the findings of this study is not publicly available due to sensitivity reasons. Interested parties can request access to the data from the corresponding author. The data are securely stored at the University Hospital Erlangen, Friedrich-Alexander-Universität Erlangen-Nürnberg, Germany.

## Abbreviations

CV: Cross-validation
HMM: Hidden Markov model
HSP: Hereditary spastic paraplegia
IMU: Inertial measurement unit
LOPO: Leave-one-participant-out
MAS: Modified Ashworth scale
MTS: Modified Tardieu scale
RF: Random forest
RoM: Range of motion
SPRS: Spastic paraplegia rating scale
SVM: Support vector machine
